# Impact of vitamin E and selenium on antioxidant capacity and lipid oxidation of cheddar cheese in accelerated ripening

**DOI:** 10.1186/s12944-018-0735-3

**Published:** 2018-04-11

**Authors:** Maryam Batool, Muhammad Nadeem, Muhammad Imran, Nabila Gulzar, Muhammad Qamar Shahid, Muhammad Shahbaz, Muhammad Ajmal, Imran Taj Khan

**Affiliations:** 1 Department of Dairy Technology, University of Veterinary and Animal Sciences, Lahore, Pakistan; 2 Institute of Home and Food Sciences, Faculty of Life Sciences, Government College University, Faisalabad, Pakistan; 3 Department of Livestock Production, University of Veterinary and Animal Sciences, Lahore, Pakistan; 4 Department of Food Science and Technology, Muhammad Nawaz Sharif University of Agriculture, Multan, Pakistan

**Keywords:** Cheddar cheese, Accelerated ripening, Vitamin E, Selenium, Oxidative stability

## Abstract

**Background:**

Ripening of cheddar cheese is a time taking process, duration of the ripening may be as long as one year. Long ripening time is a big hindrance in the popularity of cheese in developing countries. Further, energy resources in these countries are either insufficient or very expensive. Therefore, those methods of cheese ripening should be discovered which can significantly reduce the ripening time without compromising the quality characteristics of cheddar cheese. In accelerated ripening, cheese is usually ripened at higher temperature than traditional ripening temperatures. Ripening of cheddar cheese at high temperature with the addition of vitamin E and selenium is not previously studied. This investigation aimed to study the antioxidant activity of selenium and vitamin E in accelerated ripening using cheddar cheese as an oxidation substrate.

**Methods:**

The ripening of cheddar cheese was performed at 18 °C and to prevent lipid oxidation, vitamin E and selenium were used alone and in combination. The treatments were as: cheddar cheese without any addition of vitamin E and selenium (T1), cheddar cheese added with 100 mg/kg vitamin E (T_2_), 200 mg/kg vitamin E (T_3_), 800 μg/kg selenium (T_4_), 1200 μg/kg selenium (T_5_), vitamin E 100 mg/kg + 800 μg/kg selenium (T_6_) and vitamin E 200 mg/kg + 1200 μg/kg selenium (T_7_). Traditional cheddar cheese ripne ripened at 4-6 °C for 9 months was used as positive control. Cheese samples were ripened at 18 °C for a period of 12 weeks and analyzed for chemical and oxidative stability characteristics at 0, 6 and 12 weeks of storage. All these treatments were compared with a cheddar cheese without vitamin E, selenium and ripened at 4 °C or 12 weeks. Vacuum packaged cheddar cheese was ripened 18 °C for a period of 12 weeks and analyzed for chemical and oxidative stability characteristics at 0, 4 and 8 weeks of storage period.

**Results:**

Addition of Vitamin E and selenium did not have any effect on moisture, fat and protein content of cheddar cheese. After 6 weeks of ripening, total antioxidant capacity of T_1_, T_2_, T_3_, T_4_, T_5_, T_6_, T_7_ and standard cheese were 29.61%, 44.7%, 53.6%, 42.5%, 41.4%, 64.1%, 85.1% and 25.4%. After 6 weeks of ripening, reducing power of T_1_, T_2_, T_3_, T_4_, T_5_, T_6_, T_7_ and SC cheese were 14.7%, 18.1%, 26.3%, 19.2%, 25.3%, 33.4%, 40.3% and 11.6%. After 6 weeks of ripening, 1, 1-diphenyl-2-picrylhydrazyl (DPPH) free radical scavenging activity of T_6_ and T_7_ were 54.2% and 66.9%. While, DPPH free radical scavenging activity of T_1_ and standard cheese after 6 weeks of ripening were, 19.1 and 18.5%, respectively. Free fatty acids of vitamin E and selenium supplemented, non-supplemented and standard cheese were not significantly influenced from each other in 0, 6 and 12 weeks old cheddar cheese. Peroxide values of T_1_, T_2_, T_3_, T_4_, T_5_, T_6_, T_7_ and standard cheese after 6 weeks of accelerated ripening were 1.19, 1.05, 0.88, 1.25, 0.29, 0.25, 0.24 and 0.28 (MeqO_2_/kg). After 6 weeks of ripening, anisidine value of T_6_ and T_7_ were 6.55 and 6.14. Conjugated dienes of T_1_, T_2_, T_3_, T_4_, T_5_, T_6_, T_7_ and standard cheese, after 6 weeks of accelerated ripening were 0.61, 0.55, 0.42, 0.77, 0.65, 0.17, 0.15 and 0.19. After 6 weeks of accelerated ripening, concentrations unsaturated fatty acids in T_1_, T_2_, T_3_, T_4_, T_5_, T_6_, T_7_ and standard cheese decreased by18.19%, 17.45%, 16.82%, 16.19%, 12.71%, 8.48%, 6.92% and 14.71%. After 12 weeks of accelerated ripening, concentration of unsaturated fatty acids in T_1_, T_2_, T_3_, T_4_, T_5_, T_6_ and T_7_ and standard cheese decreased by 26.2%, 21.2%, 18.7%, 14.2%, 10.4%, 4.84%, 1.03% and 6.78%. Cheddar cheese samples added with vitamin E, selenium and their combinations produced more organic acids during the ripening period of 12 weeks. After 6 and 12 weeks of ripening, flavor score of T_6_ and T_7_ was better than standard ripened cheddar cheese.

**Conclusions:**

After 6 weeks of accelerated ripening, sensory characteristics of T_6_ and T_7_ were similar to cheddar cheese that was ripened at 4 °C for 9 months. Ripening time of cheddar cheese may be reduced to 6 weeks by elevated temperature (18 °C) using vitamin E and selenium as antioxidants at T_6_ and T_7_ levels.

## Background

Cheddar is recognized as a hard cheese, it is widely used in sandwiches, burgers, pizza topping and dressing of salads [1]. It frames a noteworthy extent of international trade in cheese [2]. In terms of volume and overall consumption, cheddar is the largest variety of cheese in the world [3]. Cheddar is popular all over the world due to its unique flavor characteristics that are produced during the ripening. Ripening is a process in which several biochemical changes take place that convert chalky curd to a flavorful cheese. Biochemical and metabolic processes lead to the significant changes in flavor and texture of cheese. These processes are generally referred as glycolysis, lipolysis and proteolysis. Lactose is metabolized into lactic acid, citrate diacetyl and alcohol [4]. Lipolysis and proteolysis play an important role in the breakdown of triglycerides of milk fat, generation of free fatty acids, organic acids, free amino acids, peptides, large number of flavoring compounds such as, thio-esters, alkan-2-ones, alkane-2-ols, ethyl alcohols, lactones etc. [5]. Cheddar cheese requires a long ripening time for the development of flavor and textural attributes in ripened cheese. Cheddar cheese is usually ripened for 6–12 months [6]. Few techniques are used to accelerate the ripening process of cheddar cheese, for example, utilizing elevated temperatures and enzymes [7]. The utilization of elevated temperatures for the ripening of cheese is the least complex procedure to accelerate the ripening of cheese with minimal refrigeration requiement [8]. Ripening of cheddar cheese at elevated temperature may lead to auto-oxidation, that may lead to the development of objectionable flavors, deterioration of texture and generation of potentially toxic oxidation products [9]. Temperature is a key factor in auto-oxidation of dairy and food products, oxidative stability of foods may be enhanced by antioxidants. Antioxidants are chemical substances that can neutralize and scavenge the free radicals, which are continuously produced in the body [10]. In order to prevent auto-oxidation, food and dairy products are supplemented with synthetic antioxidants, such as butylated hydroxyl anisole, butylated hydroxyl toluene and butylated hydroxyl quinone. The perceived carcinogenicity of synthetic antioxidants has led the researchers to find out the new sources of natural antioxidants and extend the application of already discovered natural antioxidants [11]. Antioxidant capacity of milk is due to sulfur containing amino acids (tyrosine and cysteine), vitamins A, E, carotenoids, enzyme systems, superoxide dismutase, catalase and glutathione peroxidase [9]. Vitamin E and selenium are important components of the antioxidant system which play important role in reducing lipid peroxidation. Vitamin E is one of the most important lipid-soluble antioxidant in milk, due to its presence in milk fat globule membrane it can prevent milk from photo-oxidation [12]. It can also act as a preventative, chain breaking antioxidant and quencher of singlet oxygen in milk [13]. Vitamin E can inhibit the activity of plasmin, a proteolytic enzyme and secondly it can directly scavenge the free radicals [14]. Vitamin E, as the primary lipid-soluble antioxidant found in foods inhibits the process of lipid peroxidation in foods and in the biological lipid-protein complexes [15]. Selenium plays an important role in the antioxidant defense system due to its requirement by the Se-dependent Glutathione Peroxidase (GSH-Px), which is involved in cellular antioxidant protection. It has been suggested that the synergistic relationship between selenium and vitamin E enhanced the antioxidant activity, because GSH-Px continues the work of vitamin E by detoxifying hydroperoxides [16]. Ripening of cheddar cheese is mandatory for the development of typical flavor and textural characteristics. Cheddar cheese is usually ripened for 6–12 months at 4-6 °C. Ripening of cheese on such a lower temperature for a fairly large period of time requires massive refrigeration. Higher ripening temperature can accelerate the metabolic processes in cheese. During rapid ripening, cheddar cheese may develop oxidized flavor. Use of natural antioxidants of milk origin in accelerated ripening of cheddar cheese is not previously investigated. This investigation aimed to study the antioxidant activity of selenium and vitamin E in accelerated ripening using cheddar cheese as an oxidation substrate.

## Methods

Cheddar cheese was prepared using the facilities of Acchha Foods, Lahore. Vitamin E (Catalogue No. 10191-41-0) and selenomethionine (Catalogue No. 3211-76-5) were purchased from Sigma Aldrich (St. Louis MO, USA). Rennet and calcium chloride were purchased from Christian Hansen, Denmark. HPLC grade chemicals, were obtained from Sigma Aldrich, USA.

### Preparation of cheddar cheese and experimental plan

Experiment was planned in a completely randomized design; each treatment was replicated three times. For the preparation of cheddar cheese, cow milk was heated to 60 °C, passed through a cream separator for the removal of excess fat. Standardized milk was used for the manufacturing of cheese. Fat, protein, lactose, minerals content, solids not fat and total solids of the standardized milk were 3.50%, 3.21%, 4.45%, 0.73%, 8.52% and 12.02%, respectively. Pasteurized in a batch pasteurizer at 68 °C for 30 min followed by cooling to 31 °C, 2% bulk starter culture comprised of *Streptococcus lactis* spp. *lactis* and *Streptococcus lactis* spp. *cremoris* were added and pre-acidification of milk was performed for 45 min, then CaCl_2_ (15 ml/100-l milk, 35% solution) and rennet (0.02%) were mixed to the cheese milk. Curd was allowed to form for 30 min then it was cut into 1.5-2 cm cubes, cooking of the curd was performed at 39 °C for 45 min, whey was drained and cheddarding was performed till pH 5.2. Batch size for every treatement of cheese was 10 kg, quanitities of vitamin E and selenomethionine were calculated for 10 kg. The detail of treatments with calculation of vitamin E and selenium is as, cheddar cheese without any addition of vitamin E and selenium (T_1_), cheddar cheese was added with 100 mg/kg vitamin E (1 g vitamin E/T_2_), 200 mg/kg vitamin E (2 g vitamin E/T_3_), 800 μg/kg selenomethionine (8 mg selenomethionine/T_4_), 1200 μg/kg selenomethionine (12 mg selenomethionine/T_5_), vitamin E 100 mg/kg + 800 μg/kg selenomethionine (1 g vitamin E + 8 mg selenomethionine/T_6_) and vitamin E 200 mg/kg + 1200 μg/kg selenomethionine (2 g vitamin E + 12 mg selenomethionine/T_7_). Traditional cheddar cheese ripne ripened at 4-6 °C for 9 months was used as positive control. Vitamin E and selenium were mixed with the milled curd at the stage of salting, followed by molding, pressing and vacuum packaging (LEVAC C46). Each treatment was replicated three times. Cheese samples were ripened at 18 °C for a period of 12 weeks and analyzed for chemical and oxidative stability characteristics at 0, 6 and 12 weeks of storage. Chemical and sensory characteritics of all the treatments were compared with a positive control i.e. cheddar cheese ripened at 4 °C for a period of 9 months. All these treatments were compared with a cheddar cheese without any addition of vitamin E and selenium and ripened at 4 °C or 12 weeks, it was written as standard cheese in this draft.

### Compositional analysis

Fat, protein and moisture content in cheese were determined at 0, 6 and 12 weeks by the standard methods [17].

### Total antioxidant capacity

Total antioxidant capacity of cheese was determined by following the method [18]. Briefly, 0.3 ml sample was mixed with 3 ml of 0.6 M sulfuric acid and 28 mM sodium phosphate and 4 mM ammonium molybdate). Test tubes were put in hot air oven at 85 °C for 90 min, absorbance of the samples was recorded at 695 nm using ascorbic acid as standard. Total antioxidant capacity was expressed in Ascorbic Acid Equivalent/g.

### Reducing power

Reducing power of cheese samples was determined according to the protocol [19]. 2.5 ml sample was mixed with equal volume of 1% potassium ferricyanide, test tubes were then incubated at 50 °C for 20 min. After this, 2.5 ml (10%) tricholoroacetic acid solution was added, tubes were centrifuged at 1000 x g for 10 min. Upper 2.5 ml layer was mixed with 2.5 ml deionized water and 0.5 ml ferric chloride (0.1%). Absorbance of the samples were recorded at 700 nm using a double beam spectrophotometer.

### 1,1-diphenyl-2-picrylhydrazyl (DPPH) free radical scavenging activity

DPPH free radical scavenging was estimated by following the method [19]. One ml sample was mixed with 1 ml (1 mM) solar solution of DPPH in methanol. Samples were incubated at room temperature for 20 min and absorbance of the samples were recorded on a double beam spectrophotometer at 517 nm and expressed in percentage.

### Antioxidant activity in linoleic acid

Sample 0.5 ml was mixed with 0.13 ml linoleic acid (99.8% in ethanol and 10 ml sodium phosphate buffer (0.2 M), volume of mixture was raised to 25 ml. Solutions were incubated in an incubator at 40 °C and thiocyanate method was used to determine the extent of oxidation. Briefly, 10 ml 75% ethanol and 0.2 ml ammonium thiocyanate and 0.2 ml ferrous chloride (20 mM in 3.5% hydrochloric acid) were mixed for three min and absorbance was recorded at 500 nm and expressed in percentage [20].

### Estimation of lipid oxidation

Free fatty acids, peroxide value, anisidine value and conjugated dienes were determined at 0, 4 and 8 weeks of ripening period [21].

### Organic acids

Grated cheese samples (5 g) were mixed with 0.1 N Phosphoric acid, homogenized at 1500 Rpm (Heidolph Diax 900, Schwabach, Germany) for the extraction of organic acids, then centrifugation was performed for 15 min at 7000 Rpm, followed by the filtration. Organic acids were determined by high performance liquid chromatography (LCM, Waters Corp., Milford, MA, USA) fitted with ion exchange chromatography column (Supelcogel C-610H, 300 × 7.8 mm, Supelco Inc., Bellefonte, PA, USA). Mobile phase consisted of phosphoric acid, flow rate was kept at 1 ml/min, 40 μl was injected and measurements were carried out at 210 nm. Internal standards of citric acid, lactic acid, formic acid and propionic acid (99% pure, Sigma Aldrich, USA) were used. Concentration of organic acids were calculated on a calibration curve, coefficient of correlations for all the measurements were not less than 0.988. Concentration of organic acids was reported as mg/kg dry matter [22].

### Sensory evaluation

Sensory evaluation of cheese samples was performed in sensory evaluation laboratory at 20 °C, in individual sensory evaluation booths. Before sensory evaluation, samples of all types of cheese were tempered to 12 °C, coded with three digit random numbers and offered in a completely randomized manner. A panel of trained judges comprising of 7 males and 3 females (age 30–50 years) performed the sensory evaluation of cheese samples for color, flavor and texture on a 9 point scale at 0, 4 and 8 week of ripening period. Sensory evaluation for every treatment was performed at least three times and coefficient of correlations was not less than R^2^ = 0.9682 [23].

### Statistical analysis

The current investigation was arranged in a Completeley Randomized Desigh (CRD). Data were analyzed by two way analysis of variance technique. For the determination of significant difference, means were compared by Tukey’s HSD Test by SAS 9.1 statistical software [24].

## Results and discussion

### Chemical composition of cheddar cheese

Addition of Vitamin E and selenium did not have any effect on moisture, fat and protein contents of cheddar cheese. Ripening of cheddar cheese at elevated temperature had a significant effect on fat and protein content of all the treatments (*p* < 0.05). After 12 weeks of accelerated ripening, fat content of T_1_, T_2_, T_3_, T_4_, T_5_, T_6_ and T_7_ decreased by 16.54%, 12.66%, 8.88%, 8.09%, 8.05%, 6.892% and 6.67%, respectively (Table 1). After 12 weeks of accelerated ripening, protein content of T_1_, T_2_, T_3_, T_4_, T_5_, T_6_ and T_7_ decreased by 11.52%, 7.68%, 7.57%, 7.42%, 7.45%, 7.16% and 7.08%. The decline in fat and protein content of cheddar cheese was due to the breakdown of lipids and protein during the ripening of cheddar cheese. The decreasing trend in protein and fat content of buffalo cheddar cheese during the ripening is reported [25]. Khan et al. [26] reported that fat and protein content of Gouda cheese decreased during the 60 days of ripening period. Ahmad et al. [27] also reported that fat and protein content of cheddar cheese decreased during the ripening period of 60 days. Folkertsma et al. [28] studied the effect of accelerated ripening on chemical composition of cheddar cheese, concentration of fat and protein decreased in both types of cheese with no effect on moisture content.

**Table 1:** Chemical Composition of Cheddar Cheese in Accelerated Ripening (18 °C)

Treatments	Ripening Period Weeks	Moisture %	Fat %	Protein %
T_1_	0	39.16 ± 0.53^a^	31.74 ± 0.42^a^	26.81 ± 0.72^a^
	6	39.11 ± 0.48^a^	29.11 ± 0.77^b^	25.47 ± 0.35^a^
	12	38.92 ± 0.67^a^	26.49 ± 0.65^c^	23.72 ± 0.66^b^
T_2_	0	39.16 ± 0.53^a^	31.74 ± 0.42^a^	26.81 ± 0.72^a^
	6	39.05 ± 0.71^a^	29.81 ± 1.19^b^	25.61 ± 1.13^a^
	12	38.77 ± 0.64^a^	27.72 ± 0.83^c^	24.63 ± 0.88^b^
T_3_	0	39.16 ± 0.53^a^	31.74 ± 0.42^a^	26.81 ± 0.72^a^
	6	39.10 ± 1.05^a^	30.92 ± 0.61^a^	25.69 ± 0.89^a^
	12	38.85 ± 0.91^a^	28.92 ± 1.37^c^	24.78 ± 0.42^b^
T_4_	0	39.16 ± 0.53^a^	31.74 ± 0.42^a^	26.81 ± 0.72^a^
	6	39.04 ± 0.59^a^	30.87 ± 1.43^a^	25.77 ± 0.93^a^
	12	38.72 ± 0.55^a^	29.17 ± 0.52^b^	24.82 ± 0.35^b^
T_5_	0	39.16 ± 0.53^a^	31.74 ± 0.42^a^	26.81 ± 0.72^a^
	6	38.81 ± 1.03^a^	30.56 ± 0.79^a^	25.81 ± 0.51^a^
	12	38.62 ± 0.43^a^	29.14 ± 0.95^b^	24.84 ± 0.59^b^
T_6_	0	39.16 ± 0.53^a^	31.74 ± 0.42^a^	26.81 ± 0.72^a^
	6	38.79 ± 0.82^a^	30.66 ± 0.97^a^	25.44 ± 1.47^a^
	12	38.65 ± 1.13^a^	29.55 ± 1.07^b^	24.89 ± 0.61^b^
T_7_	0	39.16 ± 0.53^a^	31.74 ± 0.42^a^	26.81 ± 0.72^a^
	6	38.55 ± 0.61^a^	30.95 ± 0.74^a^	25.19 ± 0.48^a^
	12	38.49 ± 0.94^a^	29.62 ± 0.88^b^	24.91 ± 0.37^b^
Standard Cheese 4 °C	0	39.16 ± 0.53^a^	31.74 ± 0.42^a^	26.81 ± 0.72^a^
	6	38.89 ± 1.37^a^	30.47 ± 0.72^a^	25.11 ± 0.54^a^
	12	38.61 ± 0.95^a^	29.44 ± 0.91^b^	24.89 ± 0.61^b^
Positive Control	Ripened at 4 °C for 9 Months	38.14 ± 0.53^a^	28.17 ± 0.69b	23.55 ± 1.39b

Within a column, means denoted by a different letter are statistically different by Tukey’s HSD Test (*p* < 0.05)

The results presented in Table 1 are the outcome of triplicate treatment and triplicate analysis (3 × 3 = 9, Mean ± SD)

T_1_: Without Vitamin E and Selenium

T_2_: 100 mg Vitamin E

T_3_: 200 mg Vitamin E

T_4_: 800 μg/kg Selenium

T_5_: 1200 μg/kg Selenium

T_6_: 100 mg Vitamin E + 800 μg/kg Selenium

T_7_: 100 mg Vitamin E + 800 μg/kg Selenium

Standard Cheese: Cheddar Cheese Ripened at 4 °C without any addition of vitamin E and selenium

Positive Control: Cheddar cheese ripened at 4 °C for 9 months

### Total antioxidant capacity (TAC)

TAC refers the antioxidant status of food substrates and measures the antioxidant reaction of food systems to scavenge/ neutralize the free radicals. TAC is a novel method for the assessment of oxidative stresses [29]. TAC is associated with oxidative stability of food, higher TAC values are usually linked with longer shelf life. Khan et al. [26] used TAC as an important parameter for the determination of antioxidant capacity of cow and buffalo milk. Results of TAC of cheddar cheese added with vitamin E and selenium are presented in Table 2. In current investigation, vitamin E, selenium and their blends were used as antioxidants in cheddar cheese exposed to accelerated ripening. Addition of vitamin E and selenium enhanced the TAC of un-ripened cheese. At zero-day, TAC of T_1_, T_2_, T_3_, T_4_, T_5_, T_6_, T_7_ and SC were 20.8%, 35.9%, 44.7%, 32.4%, 37.6%, 55.2%, 78.4% and 20.8%. TAC of standard cheese, vitamin E and selenium supplemented and non-supplemented cheese increased throughout the ripening period of 12 week. After 6 weeks of ripening, TAC of T_1_, T_2_, T_3_, T_4_, T_5_, T_6_, T_7_ and SC were 29.61%, 44.7%, 53.6%, 42.5%, 41.4%, 64.1%, 85.1% and 25.4%. TAC of 12 weeks old T_1_, T_2_, T_3_, T_4_, T_5_, T_6_, T_7_ and SC were 40.2%, 50.6%, 59.7%, 48.3%, 46.1%, 72.8%, 89.6% and 34.6%, respectively. During cheese ripening, several biochemical changes take place in cheese matrix which lead to the production of water soluble peptides that have antioxidant properties [30]. Antioxidant capacity of probiotic cheese increased with the progression of ripening [31]. Transition in antioxidant capacity of cheddar cheese in accelerated ripening is not previously reported. Supplementation of yoghurt with red ginseng increased the antioxidant capacity [32]. Surai [33] studied the synergistic effect of selenium and vitamin E on the antioxidant system of the yolk and the developing chick used in the maternal diet. The author concluded that nutritional status of the laying hen governs the efficiency of the antioxidant system throughout embryonic and early postnatal development of the offspring.

**Table 2:** Effect of Vitamin E and Selenium Supplementation on Antioxidant Capacity of Cheddar Cheese in Accelerated Ripening (18 °C)

Treatments	Ripening Period Weeks	Total Antioxidant capacity %	Reducing Power	DPPH Free Radical Scavenging Activity%	Antioxidant Activity in Linoleic Acid %
T_1_	0	20.8 ± 0.19^q^	8.22 ± 0.94^p^	17.5 ± 0.51^q^	7.81 ± 0.19^s^
	6	29.6 ± 1.22^p^	14.7 ± 0.36^n^	19.1 ± 0.72^p^	11.5 ± 0.08^q^
	12	40.2 ± 3.36^l^	16.5 ± 0.77^m^	22.4 ± 1.62^n^	16.3 ± 0.22^o^
T_2_	0	35.9 ± 0.55^o^	12.9 ± 1.05^l^	23.7 ± 1.08^m^	13.7 ± 0.28^p^
	6	44.7 ± 1.14^k^	18.1 ± 0.42^k^	26.7 ± 0.88^j^	18.4 ± 0.31^n^
	12	50.6 ± 1.59^i^	20.6 ± 0.78^j^	30.5 ± 1.43^h^	21.3 ± 0.16^m^
T_3_	0	44.7 ± 1.31^k^	23.5 ± 1.27^i^	28.2 ± 1.66^f^	17.2 ± 0.11^q^
	6	53.6 ± 2.37^h^	26.3 ± 1.44^g^	31.5 ± 1.12^h^	23.5 ± 0.46^k^
	12	59.7 ± 1.88^f^	28.4 ± 1.53^f^	33.9 ± 1.49^g^	26.4 ± 0.30^j^
T_4_	0	32.4 ± 1.6^1^	14.6 ± 1.69^m^	20.3 ± 0.61^g^	22.4 ± 0.72^l^
	6	42.5 ± 1.42^l^	19.2 ± 1.15^n^	24.5 ± 0.79^l^	28.9 ± 1.44^i^
	12	48.3 ± 2.77^i^	24.7 ± 1.38^h^	27.7 ± 0.83^i^	32.8 ± 1.57^g^
T_5_	0	37.6 ± 1.1^n^	22.5 ± 0.91^i^	21.9 ± 1.23^j^	24.4 ± 0.93^g^
	6	41.4 ± 1.71^l^	25.3 ± 0.57^h^	25.8 ± 1.11^k^	29.3 ± 1.29^h^
	12	46.1 ± 1.94^j^	26.9 ± 0.95^g^	27.4 ± 1.56^i^	35.6 ± 1.75^f^
T_6_	0	55.2 ± 2.39^g^	30.5 ± 0.32^e^	41.5 ± 1.89^f^	37.9 ± 2.33^e^
	6	64.1 ± 1.77^e^	33.4 ± 1.61^d^	54.2 ± 1.97^e^	45.4 ± 1.99^e^
	12	72.8 ± 3.46^d^	35.2 ± 1.51^c^	62.5 ± 2.27^c^	53.4 ± 2.82^d^
T_7_	0	78.4 ± 2.91^c^	37.4 ± 1.43^b^	58.3 ± 3.19^d^	56.7 ± 1.16^c^
	6	85.1 ± 1.66^b^	40.3 ± 1.31^a^	66.9 ± 2.88^b^	67.9 ± 3.36^b^
	12	89.6 ± 1.39^a^	41.7 ± 1.03^a^	82.8 ± 4.36^a^	75.6 ± 2.88^a^
Standard Ripening 4 °C	0	20.8 ± 0.19^q^	8.22 ± 0.94^p^	17.5 ± 0.51^q^	7.81 ± 0.19^s^
	6	25.4 ± 0.48^p^	11.6 ± 0.22^o^	18.5 ± 0.38^p^	9.27 ± 0.19^r^
	12	34.6 ± 0.75^o^	19.8 ± 0.66^j^	21.2 ± 0.0.25^o^	13.2 ± 0.44^p^
Positive Control	Ripened at 4 °C for 9 Months	49.7 ± 1.54^i^	15.9 ± 0.73^m^	32.9 ± 1.19 g	23.1 ± 0.87^l^

Within a column, means denoted by a different letter are statistically different by Tukey’s HSD Test (*p* < 0.05)

The results presented in Table 2 are the outcome of triplicate treatment and triplicate analysis (3 × 3 = 9; Mean ± SD)

### Reducing power

Vitamin E and selenium increased the reducing power of un-ripened cheddar cheese. At zero day, reducing power of T_1_, T_2_, T_3_, T_4_, T_5_, T_6_, T_7_ and SC cheese were 8.22%, 12.9%, 23.5%, 14.6%, 22.5%, 30.5%, 37.4% and 8.22% (Table 2). Reducing power of standard cheese, vitamin E and selenium supplemented and non-supplemented cheese increased throughout the ripening period of 12 week. After 6 weeks of ripening, reducing power of T_1_, T_2_, T_3_, T_4_, T_5_, T_6_, T_7_ and SC cheese were 14.7%, 18.1%, 26.3%, 19.2%, 25.3%, 33.4%, 40.3% and 11.6%. Antioxidant substances in milk can scavenge superoxide radicals, hydroxyl radicals and peroxide radicals [19]. Supplementation of sheep butter with vitamin E considerably improved the oxidative stability [34]. Oxidative stresses can lead to cancer, diabetes, accelerated ageing and breakdown of biochemical compounds such as, lipids and proteins [35]. Demand of food containing natural antioxidants is increasing all over the world, researchers are trying to develop functional food containing natural antioxidants. Saldamli [36] supplemented Turkish white cheese with selenium to assess their recoveries in cheese, they recorded that recovery of selenium in supplemented cheese was about 71%. This is the pioneer study in which the role of selenium and vitamin as E as antioxidants were determined in accelerated cheese ripening. United States Dietary Guidelines suggest to intake 50 μg/day, maximum intake of selenium should be less than 200 μg/day. Antioxidant and antitumor activities of selenium have been reported in literature, intake of selenium reduced the risks of heart diseases, cystic fibrosis and cancer [37]. Antioxidant activity of selenium for the inhibition of superoxide dismutase is scientifically proven [38]. Glutathione and selenium enhanced the functional value, antioxidant capacity of milk [39]. Vitamin E and selenium efficiently inhibited the lipid oxidation in vacuum packaged lamb meat [40].

### 1, 1-diphenyl-2-picrylhydrazyl (DPPH) free radical scavenging activity

DPPH assay is widely used for the antioxidant characterization of dairy products. Ullah et al. [41] used DPPH assay to study the antioxidant capacity of chia oil supplemented cheddar cheese. Supplementation of cheddar cheese with selenium and vitamin E considerably improved the antioxidant capacity of freshly prepared cheddar cheese. At zero day of ripening, highest antioxidant capacity was recorded in cheese supplemented with 100 mg Vitamin E + 800 μg/kg Selenium. DPPH free radical scavenging activity went on increasing in all samples of cheese. However, DPPH free radicals scavenging activity of selenium and vitamin E supplemented samples in accelerated ripening were higher than non-supplemented and standard ripened cheese. Antioxidant capacity of cheese remarkably increased when selenium and vitamin E were used in combination e.g. antioxidant capacity of cheese supplemented with 100 mg Vitamin E + 800 μg/kg Selenium and 2100 mg Vitamin E + 1200 μg/kg Selenium after 6 weeks of accelerated ripening were 54.2% and 66.9%. While the antioxidant capacity of non-supplemented and standard ripened cheese after 6 weeks of ripening were 19.1% and 18.5% (Table 2). Antioxidant capacity of cheese supplemented with 100 mg Vitamin E + 800 μg/kg Selenium and 2100 mg Vitamin E + 1200 μg/kg Selenium after 12 weeks of accelerated ripening were 62.5% and 82.8%. While the antioxidant capacity of non-supplemented and standard ripened cheese after 6 weeks of ripening were 19.1% and 18.5%. DPPH free radical scavenging activity of mature cheese was greater than fresh cheese [42]. Barać et al. [43] reported that antioxidant capacity of cheese went on increasing during the ripening period. Antioxidant effect of vitamin E and selenium on omega-3 enriched poultry meat was investigated. Thiobarbituric acid value decreased with the addition of vitamin E and selenium [44].

### Antioxidant activity in linoleic acid (ALA)

Supplementation of cheddar cheese with vitamin E and selenium significantly improved the ALA of freshly prepared cheddar cheese. Combination of selenium and vitamin E considerably enhanced ALA of un-ripened cheddar cheese. ALA of T_5_ (100 mg vitamin E and 800 μg/kg selenium) and T_6_ (200 mg vitamin E and 1200 μg/kg selenium) was 37.9% and 56.7%. ALA of all types of cheese samples increased during the ripening period of 12 weeks, however, cheese samples exposed to accelerated ripening had higher ALA than cheese sample ripened at 4 °C. Similarly, cheese samples supplemented with combination of vitamin E and selenium exhibited highest ALA, for example, antioxidant activity of 6 weeks old T_6_ and T_7_ were 45.4% and 67.9%. ALA of 12 weeks old T_6_ and T_7_ were 53.4% and 75.6%. Cheese samples having ALA had lower peroxide value, anisidine value and conjugated dienes with better sensory score. Rashidinejad et al. [45] monitored the antioxidant activity of cheddar cheese added with tea extract, antioxidant activity of cheese increased during the ripened. Branciari et al. [46] reported that phenolic compounds of rosemary leaf significantly improved the antioxidant capacity of cheese and efficiently inhibited the lipid oxidation. Addition of catechin, improved the antioxidant capacity of cheese [47]. Supplementation of cow feed with vitamin E and selenium improved the antioxidant status of blood [48].

### Lipid oxidation

#### Free fatty acids

In cheese, free fatty acids are produced as a result of hydrolysis of triglycerides, catalytic factors include moisture content, lipases, storage temperature, metal ions etc. [49]. Free fatty acid plays an important role in the development of flavor in cheese [50]. Supplementation of vitamin E and selenium did not have any impact on free fatty acids content of cheddar cheese at 0, 6 and 12 weeks of ripening (Table 3). Free fatty acids of trans free margarine increased during the three months storage time [51]. Hydrolysis of the triglyerides is the major reason for the production of free fatty acids with no connection with auto-oxidation [52]. No correlation was established between free fatty acids and antioxicant [53]. Usually the concentration of free fatty acids increase during cheese ripening. Concentration of free fatty acids in mature cheese was more than fresh cheese [54]. Free fatty acid may accelerate the auto-oxidation in fats by speeding up breakdown of peroxides to oxidation products [55]. Supplmentatio of hen diet with vitamin E and selenium significantly rasied the antioxidant characteritics of eggs [56].

**Table 3:** Effect of Selenium and Vitamin E Supplementation on Oxidative Stability of Cheddar Cheese in Accelerated Ripening (18 °C)

Treatments	Ripening Period Weeks	Free Fatty Acids (Oleic Acid) %	Peroxide Value (MeqO_2_/kg)	Anisidine Value	Conjugated Dienes (^1%^ε1cm [λ232])
T_1_	0	0.11 ± 0.02^f^	0.22 ± 0.03^j^	5.81 ± 0.14^l^	0.08 ± 0.01^j^
	6	0.22 ± 0.04^c^	1.12 ± 0.07^e^	9.73 ± 0.08^g^	0.61 ± 0.03^f^
	12	0.51 ± 0.07^a^	2.85 ± 0.12^a^	22.5 ± 0.59^a^	2.17 ± 0.09^a^
T_2_	0	0.11 ± 0.02^f^	0.22 ± 0.03^j^	5.81 ± 0.14^l^	0.08 ± 0.01^j^
	6	0.24 ± 0.03^c^	1.05 ± 0.14^e^	10.6 ± 0.88^f^	0.55 ± 0.02^g^
	12	0.48 ± 0.05^a^	2.51 ± 0.09^a^	20.7 ± 1.10^b^	1.63 ± 0.14^c^
T_3_	0	0.11 ± 0.02^f^	0.22 ± 0.03^j^	5.81 ± 0.14^l^	0.08 ± 0.01^j^
	6	0.19 ± 0.03^b^	0.88 ± 0.16^f^	9.4 ± 0.65^g^	0.42 ± 0.03^h^
	12	0.49 ± 0.06^a^	1.61 ± 0.19^c^	17.3 ± 0.40^d^	1.39 ± 0.07^e^
T_4_	0	0.11 ± 0.02^f^	0.22 ± 0.03^j^	5.81 ± 0.14^l^	0.08 ± 0.01^j^
	6	0.23 ± 0.01^c^	1.25 ± 0.15^d^	9.14 ± 0.31^h^	0.77 ± 0.13^e^
	12	0.52 ± 0.04^a^	2.18 ± 0.18^b^	18.6 ± 0.92^c^	1.85 ± 0.06^b^
T_5_	0	0.11 ± 0.02^f^	0.22 ± 0.03^j^	5.81 ± 0.14^l^	0.08 ± 0.01^j^
	6	0.18 ± 0.01^c^	0.29 ± 0.06^j^	8.97 ± 1.24^h^	0.65 ± 0.05^f^
	12	0.50 ± 0.07^a^	0.51 ± 0.02^g^	16.2 ± 1.55^e^	1.49 ± 0.12^d^
T_6_	0	0.11 ± 0.02^f^	0.22 ± 0.03^j^	5.81 ± 0.14^l^	0.08 ± 0.01^j^
	6	0.21 ± 0.06^b^	0.25 ± 0.02^j^	6.55 ± 0.48^j^	0.17 ± 0.02^i^
	12	0.53 ± 0.07^a^	0.42 ± 0.04^h^	8.91 ± 0.69^h^	0.41 ± 0.04^h^
T_7_	0	0.11 ± 0.02^f^	0.22 ± 0.03^j^	5.81 ± 0.14^l^	0.08 ± 0.01j
	6	0.19 ± 0.04^b^	0.24 ± 0.05^j^	6.14 ± 0.37^k^	0.15 ± 0.02i
	12	0.49 ± 0.03^a^	0.28 ± 0.02^j^	7.75 ± 0.66^i^	0.34 ± 0.01^h^
Standard Ripening 4 °C	0	0.11 ± 0.02^f^	0.22 ± 0.03^j^	5.81 ± 0.14^k^	0.08 ± 0.01^j^
	6	0.16 ± 0.01^e^	0.27 ± 0.02^j^	6.05 ± 0.08^j^	0.19 ± 0.03^i^
	12	0.31 ± 0.04^b^	0.35 ± 0.01^i^	8.11 ± 0.17^h^	0.39 ± 0.04^h^
Positive Control	Ripened at 4 °C for 9 Months	0.47 ± 0.02a	0.45 ± 0.05^h^	7.92 ± 0.06^i^	0.44 ± 0.01^h^

Within a column, means denoted by a different letter are statistically different by Tukey’s HSD Test (*p* < 0.05)

The results presented in Table 3 are the outcome of triplicate treatment and triplicate analysis (3 × 3 = 9; Mean ± SD)

### Peroxide value

Peroxide value describes the degree of oxidation previously taken place in fats and oils, it is one of the most commonly used chemical marker in quality control labs and edible oil industries to determine the oxidation status of fats and oils [57]. Peroxide value greatly depends upon the storage conditions, temperature has a major effect on peroxide value of fats [58]. In current investigation, cheddar cheese was ripened at higher temperature (18 °C). Peroxide value of vitamin E and selenium supplemented cheddar cheese, non-supplemented and standard cheese went on increasing throughout the ripening period of 12 weeks. Supplementation of cheddar cheese with vitamin E and selenium alone and in combination significantly inhibited the auto-oxidation in cheese samples. After 6 weeks of accelerated ripening, peroxide values of T_1_, T_2_, T_3_, T_4_, T_5_, T_6_, T_7_ and standard cheese were 1.19, 1.05, 0.88, 1.25, 0.29, 0.25, 0.24 and 0.28 (MeqO_2_/kg). After 12 weeks of accelerated ripening, peroxide value of T_1_, T_2_, T_3_, T_4_, T_5_, T_6_ and standard cheese were 2.85, 2.51, 1.61, 2.18, 0.51, 0.42, 0.28 and 0.35(MeqO_2_/kg). Peroxide value may be used to anticipate the oxidative stability/ shelf life of fat rich foods, lower peroxide value is usually linked with excellent storage stability [53]. Peroxide value and concentration of antioxidants were strongly correlated (R^2^ = 0.992). It is worth mentioning that peroxide value was strongly correlated with flavor score, treatments having higher peroxide value had lower flavor score. Khan et al. [59] studied the effect of mango kernel oil on oxidative stability of Gouda cheese, natural antioxidant substances efficiently inhibited the auto-oxidation in cheese. Natural antioxidants of mango kernel oil altered the auto-oxidation of milk fat, protected fatty acids from oxidation [58]. Peroxide value of butter and ice cream increased during the storage period [60]. Combined supplementation of vitamin E and selenium considerably improved the lipid stability of veal [61].

### Anisidine value

Anisidine value is hundred times the optical density of a chemical reaction taking place between 2-alkenals and anisidine. It is a good measure of secondary and tertiary oxidation products [62]. Anisidine value is usually not used during the cheese related studies, in current investigation, it was included as an important parameter to measure the magnitude of secondary and tertiary oxidation products in accelerated cheese ripening. Vitamin E and selenium supplemented cheese samples revealed lower anisidine value than non-supplemented cheese samples (Table 3). After 6 weeks of accelerated ripening at 18 °C, anisidine vaue of T_6_ and T_7_ was 6.55 and 6.14. After 12 weeks of accelerated ripening anisidine values of T_6_ and T_7_ and T_1_ were 8.91, 7.75 and 22.5. Anisidine value of T_6_ and T_7_ was less than the standard limit of 10. The lower anisidine value in vitamin E and selenium supplemented cheese was due to their antioxidant activity in cheese matrix. Anisidine value is extensively used for the assessment of oxidation products in butter, butter oil, cottonseed oil, ice cream and cheese [52, 58, 63, 64]. Soliman et al. [65] stated the synergistic effect of vitamin E and selenium on some physiological and productive characteristics of ewes and their lambs. There is improved growth performance, immune responses and viability as a result of favorable signs in their physiological reactions.

### Conjugated dienes (CD)

Results of specific extinction coefficients of different cheese samples measured at 230 nm are presented in Table 3. CD measure the extent of primary oxidation products produced as a result of auto-oxidation in fats and oils [66]. Supplementation of cheese with vitamin E and selenium considerably inhibited the formation of CD in accelerated ripening. CD of T_1_, T_2_, T_3_, T_4_, T_5_, T_6_ and T_7_, after 6 weeks of accelerated ripening were 0.61, 0.55, 0.42, 0.77, 0.65, 0.17, 0.15 and 0.19. While, CD of 6 weeks old standard cheese was 0.19. After 12 weeks of accelerate ripening, CD of T_1_, T_6_ and T_7_ was 2.17, 0.41 and 0.34. After 12 weeks, CD of T_7_ and standard ripened cheese were not significantly influence from each other. Few studies have been reported regarding the inhibition of oxidative breakdown in dairy products. *Moringa oleifera* leaf extract significantly inhibited CD of butter during the storage period of 90 days [52]. Sesame (*Sesamum indicum* L.) cake extract inhibited the formation of CD in olein based butter. CD of olein based ice cream increased during the storage period of 180 days [64]. Conjugated dienes of sunflower oil increased during the storage period [35]. Chatha et al. [67] also described an increasing trend in the magnitude of conjugated dienes, when canola oil was subjected to storage. Limited information is available regarding the antioxidant activity of selenium in dairy products. Concentration of oxidation products increased during the one-year storage of vegetable oil blends [68]. Oxidation status of dairy products changed during the storage [69]. Habibian et al. [70] investigated the effects of dietary selenium and vitamin E on growth performance, meat yield, and selenium content and lipid oxidation of breast meat of broilers reared under heat stress. They concluded that supplementation of selenium and vitamin E improved the antioxidant capacity of breast meat.

### Fatty acid profile

Fatty acid profile is one of the most significant parameter to determine the oxidation status of fats and oils [71]. In current investigation, effect of vitamin E and selenium supplmenttion on fatty acid profile of cheddar cheee was determined in accelerated ripening. For the assessment of oxidative stability of dairy products, fatty acid profile is used, the techniques of accelerated oxidation are traditionally not employed to forecast the oxidative stability of cheese and other dairy products. Nadeem et al. [53] used fatty acid profile as an important parameter to adjudge the antioxidant capacity of whey butter. Ullah et al. [41] while studying the effect of chia oil on antioxidant capacity of cheddar cheese used fatty acid profile as a marker of oxidative stability. Addition of selenium and vitamin E alone and in combination did not have any effect on fatty acid profile of fresh cheese. Fatty acid profile of all the treatments and standard cheese considerably changed during the rapid and traditional ripening. However, the magnitude of transition was dependent upon the ripening temperature, type and dose of antioxidant added in cheese. Concentration of short-chain, medium-chain and long-chain fatty acids decreased during the ripening period of 12 weeks. After 6 weeks of accelerated ripening, concentrations short-chain fatty acids in T_1_, T_2_, T_3_, T_4_, T_5_, T_6_, T_7_ and standard cheese decreased by 22.55%, 20.10%, 19.2%, 18.29%, 5.25%, 4.89%, 4.89%, 3.08% (Table 4). After 6 weeks of accelerated ripening, concentrations medium-chain fatty acids in T_1_, T_2_, T_3_, T_4_, T_5_, T_6_, T_7_ and standard cheese decreased by10.59%, 9.27%, 6.49%, 5.69%, 3.15%, 2.02%, 1.41% and 5.95%. After 6 weeks of accelerated ripening, concentrations unsaturated fatty acids in T_1_, T_2_, T_3_, T_4_, T_5_, T_6_, T_7_ and standard cheese decreased by18.19%, 17.45%, 16.82%, 16.19%, 12.71%, 8.48%, 6.92% and 14.71%. After 12 weeks of accelerated ripening, concentration of short-chain fatty acids in T_1_, T_2_, T_3_, T_4_, T_5_, T_6_ and T_7_ decreased by 33.5%, 30.7%, 17.1%, 9.94%, 8.13%, 1.9% and 1.08%, from their initial values. Concentration of short-chain fatty acids in standard cheese decreased by 3.17%, from the initial value. After 12 weeks of accelerated ripening, concentration of medium-chain fatty acids in T_1_, T_2_, T_3_, T_4_, T_5_, T_6_ and T_7_ and standard ripened cheddar cheese decreased by 15.4%, 13.4%, 11.02%, 7.34%, 4.73%, 0.81%, 0.55% and 10.79%, respectively. After 12 weeks of accelerated ripening, concentration of unsaturated fatty acids in T_1_, T_2_, T_3_, T_4_, T_5_, T_6_ and T_7_ and standard cheese decreased by 26.2%, 21.2%, 18.7%, 14.2%, 10.4%, 4.84%, 1.03% and 6.78%. Unsaturated fatty acids are more susceptible to auto-oxidation as compared to saturated fatty acids, oxidation rate of oleic acid, linoleic acid and linoleic acid is 15, 25 and 35 times higher than stearic acid [71]. Vitamin E and selenium when used in combination (T_6_ and T_7_) considerably inhibited the auto-oxidation of unsaturated fatty acids and formation of oxidation products. Minimum values for conjugated dienes and anisidine value were observed for T_6_ and T_7_ after 12 weeks of accelerated ripening. Arif et al. [72] described that concentration of short-chain, medium-chain and long-chain fatty acids decreased in butter oil during the storage period of 2 months. Nadeem et al. [52] observed that antioxidants of *Moringa oleifera* leaves efficiently inhibited the lipid oxidation in butter oil, treatments added with natural antioxidant revealed minimum changes in fatty acid profile. Rayman et al. [73] studied the effect of various levels of selenium supplementation on total cholesterol and HDL, supplementation of selenium at 100–300 mcg/day decreased total cholesterol and increased HDL. Neutracueticals may be very useful to prevent/ correct dyslipidemia [74].

**Table 4:** Effect of Selenium and Vitamin E on Fatty Acid Profile of Cheddar Cheese in Accelerated Ripening (18 °C)

Fatty Acid	Weeks	T_1_	T_2_	T_3_	T_4_	T_5_	T_6_	T_7_	Standard Ripening 4 °C	9 Months Old Cheese*
C_4:0_	0	3.82 ± 0.04^a^	3.82 ± 0.04^a^	3.82 ± 0.04^a^	3.82 ± 0.04^a^	3.82 ± 0.04^a^	3.82 ± 0.04^a^	3.82 ± 0.04^a^	3.82 ± 0.04^a^	3.42 ± 0.08^b^
	6	3.11 ± 0.07^b^	3.27 ± 0.01^b^	3.31 ± 0.05^b^	3.34 ± 0.02^b^	3.78 ± 0.06^b^	3.79 ± 0.02^a^	3.80 ± 0.03^a^	3.65 ± 0.09^a^	
	12	2.61 ± 0.03^c^	2.64 ± 0.01^c^	2.69 ± 0.02^c^	2.71 ± 0.24^c^	3.35 ± 0.07^b^	3.57 ± 0.05^a^	3.59 ± 0.13^a^	3.62 ± 0.02^a^	
C_6:0_	0	2.26 ± 0.07^a^	2.26 ± 0.07^a^	2.26 ± 0.07^a^	2.26 ± 0.07^a^	2.26 ± 0.07^a^	2.26 ± 0.07^a^	2.26 ± 0.07^a^	2.26 ± 0.07^a^	2.15 ± 0.09b
	6	1.71 ± 0.01^c^	1.74 ± 0.05^c^	1.75 ± 0.04^c^	1.78 ± 0.06^c^	2.12 ± 0.01^b^	2.08 ± 0.02^b^	2.10 ± 0.08^b^	2.19 ± 0.10^b^	
	12	0.54 ± 0.09^f^	0.59 ± 0.03^f^	0.71 ± 0.09^e^	0.85 ± 0.11^e^	1.11 ± 0.03^d^	1.21 ± 0.01^d^	1.27 ± 0.04^d^	2.15 ± 0.03^b^	
C_8:0_	0	1.61 ± 0.05^a^	1.61 ± 0.05^a^	1.61 ± 0.05^a^	1.61 ± 0.05^a^	1.61 ± 0.05^a^	1.61 ± 0.05^a^	1.61 ± 0.05^a^	1.61 ± 0.05^a^	1.52 ± 0.06^b^
	6	1.14 ± 0.02^d^	1.16 ± 0.02^c^	1.18 ± 0.01^c^	1.20 ± 0.09^c^	1.44 ± 0.03^b^	1.48 ± 0.02^b^	1.49 ± 0.04^b^	1.59 ± 0.02^b^	
	12	0.35 ± 0.11^g^	0.65 ± 0.02^f^	0.64 ± 0.03^f^	0.97 ± 0.16^e^	1.18 ± 0.12^b^	1.36 ± 0.09^b^	1.38 ± 0.13^b^	1.55 ± 0.17^b^	
C_10:0_	0	3.35 ± 0.14^a^	3.35 ± 0.14^a^	3.35 ± 0.14^a^	3.35 ± 0.14^a^	3.35 ± 0.14^a^	3.35 ± 0.14^a^	3.35 ± 0.14^a^	3.35 ± 0.14^a^	2.29 ± 0.02^b^
	6	2.59 ± 0.11^b^	2.65 ± 0.05^b^	2.68 ± 0.07^b^	2.70 ± 0.06^b^	3.12 ± 0.04^a^	3.15 ± 0.02^a^	3.11 ± 0.01^a^	3.27 ± 0.08^a^	
	12	1.18 ± 0.06^f^	1.21 ± 0.08^f^	1.44 ± 0.04^e^	1.91 ± 0.08^d^	2.16 ± 0.18^c^	2.29 ± 0.05^c^	2.32 ± 0.06^c^	3.21 ± 0.20^a^	
C_12:0_	0	4.18 ± 0.15^a^	4.18 ± 0.15^a^	4.18 ± 0.15^a^	4.18 ± 0.15^a^	4.18 ± 0.15^a^	4.18 ± 0.15^a^	4.18 ± 0.15^a^	4.18 ± 0.15^a^	3.72 ± 0.04c
	6	3.54 ± 0.03^d^	3.61 ± 0.07^d^	3.67 ± 0.06^c^	3.71 ± 0.09^c^	3.91 ± 0.02^b^	3.95 ± 0.07^b^	3.98 ± 0.05^b^	4.12 ± 0.12^a^	
	12	1.98 ± 0.12^h^	2.03 ± 0.05^h^	2.28 ± 0.09^g^	2.49 ± 0.04^f^	2.81 ± 0.03^f^	3.14 ± 0.09^e^	3.15 ± 0.03^e^	4.09 ± 0.13^a^	
C_14:0_	0	11.53 ± 0.44^a^	11.53 ± 0.44^a^	11.53 ± 0.15^a^	11.53 ± 0.15^a^	11.53 ± 0.15^a^	11.53 ± 0.15^a^	11.53 ± 0.15^a^	11.53 ± 0.15^a^	9.58 ± 0.28c
	6	9.28 ± 0.19^c^	9.44 ± 0.25^c^	10.55 ± 0.13^b^	10.61 ± 0.21^b^	10.91 ± 0.43^b^	11.27 ± 0.56^a^	11.32 ± 0.26^a^	11.41 ± 0.64^a^	
	12	8.32 ± 0.71^d^	8.82 ± 0.31^d^	9.45 ± 0.34^c^	9.54 ± 0.77^c^	10.14 ± 0.19^b^	10.49 ± 0.51^b^	10.44 ± 0.32^b^	11.17 ± 0.43^a^	
C_16:0_	0	28.76 ± 1.25^a^	28.76 ± 1.25^a^	28.76 ± 1.25^a^	28.76 ± 1.25^a^	28.76 ± 1.25^a^	28.76 ± 1.25^a^	28.76 ± 1.25^a^	28.76 ± 1.25^a^	23.19 ± 0.42^e^
	6	26.74 ± 0.48^c^	27.13 ± 0.39^b^	27.25 ± 0.73^b^	27.56 ± 0.77^b^	28.21 ± 0.95^a^	28.39 ± 0.37^a^	28.55 ± 0.73^a^	28.63 ± 0.17^a^	
	12	22.14 ± 0.59^f^	23.55 ± 0.72^e^	23.92 ± 0.80^e^	23.55 ± 0.93^e^	24.79 ± 1.19^d^	26.55 ± 0.61^c^	27.64 ± 1.11^b^	28.33 ± 1.36^a^	
C_18:0_	0	9.42 ± 0.63^a^	9.42 ± 0.63^a^	9.42 ± 0.63^a^	9.42 ± 0.63^a^	9.42 ± 0.63^a^	9.42 ± 0.63^a^	9.42 ± 0.63^a^	9.42 ± 0.63^a^	7.11 ± 0.13^d^
	6	8.62 ± 0.32^c^	8.71 ± 0.16^c^	8.92 ± 0.16^c^	8.94 ± 0.41^c^	9.16 ± 0.07^b^	9.19 ± 0.22^a^	9.27 ± 0.55^a^	9.22 ± 0.18^a^	
	12	7.12 ± 0.37^d^	7.25 ± 0.21^d^	7.31 ± 0.47^d^	8.35 ± 0.11^c^	8.61 ± 0.81^c^	9.27 ± 0.46^a^	9.36 ± 0.71^a^	8.91 ± 0.39^b^	
C_18:1_	0	23.59 ± 0.51^a^	23.59 ± 0.51^a^	23.59 ± 0.51^a^	23.59 ± 0.51^a^	23.59 ± 0.51^a^	23.59 ± 0.51^a^	23.59 ± 0.51^a^	23.59 ± 0.51^a^	20.13 ± 0.69d
	6	21.38 ± 0.24^c^	21.49 ± 0.62^c^	21.56 ± 0.40^c^	21.66 ± 0.77^c^	21.82 ± 0.32^c^	22.67 ± 0.25^b^	22.87 ± 0.44^b^	23.39 ± 0.84^a^	
	12	17.36 ± 0.94^g^	18.41 ± 1.02^f^	18.78 ± 0.93^f^	19.47 ± 0.51^e^	20.55 ± 0.78^d^	21.45 ± 0.95^c^	22.47 ± 0.35^b^	22.94 ± 0.79b	
C_18:2_	0	1.98 ± 0.13^a^	1.98 ± 0.13^a^	1.98 ± 0.13^a^	1.98 ± 0.13^a^	1.98 ± 0.13^a^	1.98 ± 0.13^a^	1.98 ± 0.13^a^	1.98 ± 0.13^a^	1.22 ± 0.06d
	6	0.59 ± 0.06^g^	0.71 ± 0.02^f^	0.72 ± 0.01^f^	0.75 ± 0.04^f^	1.52 ± 0.09^c^	1.72 ± 0.05^b^	1.91 ± 0.02^a^	1.81 ± 0.16^b^	
	12	0.27 ± 0.09^i^	0.38 ± 0.06^h^	1.09 ± 0.26^e^	1.27 ± 0.08^d^	1.47 ± 0.05^c^	1.57 ± 0.22^c^	1.91 ± 0.36^a^	1.71 ± 0.31^b^	
C_18:3_	0	0.42 ± 0.02^a^	0.42 ± 0.02^a^	0.42 ± 0.02^a^	0.42 ± 0.02^a^	0.42 ± 0.02^a^	0.42 ± 0.02^a^	0.42 ± 0.02^a^	0.42 ± 0.02^a^	0.19 ± 0.03d
	6	0.11 ± 0.01^e^	0.08 ± 0.03^f^	0.17 ± 0.04^d^	0.21 ± 0.01^c^	0.22 ± 0.03^c^	0.31 ± 0.01^b^	0.34 ± 0.01^b^	0.24 ± 0.01^c^	
	12	Not Detected	Not Detected	Not Detected	0.17 ± 0.01^c^	0.19 ± 0.07^c^	0.18 ± 0.05^c^	0.27 ± 0.01^a^	0.22 ± 0.03^b^	

Within a row, means denoted by a different letter are statistically different by Tukey’s HSD Test (*p* < 0.05)

The results presented in Table 4 are the outcome of triplicate treatment and triplicate analysis (3 × 3 = 9; Mean ± SD)

*Cheddar cheese ripened at 4 °C for 9 months: Positive Control

### Organic acids

Ripening of cheddar cheese is a complex phenomenon, several biochemical changes take place that influence the flavor and texture perspectives of the cheese. In current investigation, effect of accelerated ripening, vitamin E and selenium was recorded on the production of organic acids. Accelerated ripening, vitamin E and selenium significantly influenced the production of organic acid. After 12 weeks of ripening at 4 °C, cheddar cheese revealed the lowest concentration of organic acids. Concentrations of formic acid, pyruvic acid, lactic acid, acetic acid and citric acid in 12 weeks old standard ripened cheddar cheese (4 °C) were 519, 88, 8463, 262 and 649 ppm, respectively. Cheddar cheese samples added with vitamin E, selenium and their combinations produced more organic acids during the ripening period of 12 weeks. Concentrations of formic acid, pyruvic acid, lactic acid, acetic acid and citric acid in 12 weeks old T_1_ were 627, 115, 9253, 311 and 691 ppm. Concentrations of formic acid, pyruvic acid, lactic acid, acetic acid and citric acid in 12 weeks old T_2_ were 659, 128, 9642, 339 and 728 ppm. Concentrations of formic acid, pyruvic acid, lactic acid, acetic acid and citric acid in 12 weeks old T_3_ were 676, 144, 10,118, 362 and 814 ppm. Concentrations of formic acid, pyruvic acid, lactic acid, acetic acid and citric acid in 12 weeks old T_4_ were 697, 155, 10,237, 391 and 849 ppm. Concentrations of formic acid, pyruvic acid, lactic acid, acetic acid and citric acid in 12 weeks old T_5_ were 751, 192, 10,663, 427 and 914 ppm. Concentrations of formic acid, pyruvic acid, lactic acid, acetic acid and citric acid in 120 days old T_6_ were 923, 269, 125,539, 633 and 1162 ppm. Concentrations of formic acid, pyruvic acid, lactic acid, acetic acid and citric acid in 12 weeks old T_7_ were 1156, 295, 12,987, 751 and 1264 ppm. Hough et al. [75] observed that flavor characteristics of Reggianio cheese improved with the production of organic acids. Accelerated ripening boosted the production of organic acids [76]. However, the production of organic acids in accelerated ripening of cheddar cheese in presence of antioxidants is not previously studied.

### Sensory characteristics

Addition of vitamin E and Selenium alone and in combination did not have any effect significant effect on color, flavor and texture of fresh cheddar cheese. Color score of all the treatments at 0, 6 and 12 weeks of ripening were not different from the standard cheese. Ripening temperature significantly affected the sensory characteritics of cheese, cheddar cheese exposed to accelerate ripening ripened earlier than standard cheese. Flavor score of 6 weeks old T_2_, T_3_, T_4_ and T_5_ were not different from 12 weeks old standard ripened cheddar cheese (Table 5). After 6 weeks of accelerated ripening, flavor score of T_6_ and T_7_ and positive control (cheddar cheese ripened at 4 °C for 9 months) was not different from each other. Flvor score was closely related with peroxide value (R^2^ = 0.9987). A strong correlation between peroxide value and flavor characteristics of dairy product is recorded by [64]. Nadeem et al. [53] observed that flavor score of whey butter decreased with the rise in peroxide value. Addition of sesame cake extract as antioxidant in olein based butter considerably inhibited the auto-oxidation [53]. Flavor score of fresh and 90 days old cheese was different from each other [54].

**Table 5:** Effect of Selenium and Vitamin E Supplementation on Sensory Characteristics of Cheddar Cheese in Accelerated Ripening (18 °C)

Treatments	Ripening Period Weeks	Color	Flavor	Texture
T_1_	0	8.2 ± 0.16^a^	6.1 ± 0.14^e^	6.5 ± 0.16^d^
	6	8.3 ± 0.11^a^	6.7 ± 0.21^c^	6.2 ± 0.09^d^
	12	8.0 ± 0.05^a^	5.5 ± 0.16^f^	5.8 ± 0.05^e^
T_2_	0	8.1 ± 0.24^a^	6.3 ± 0.08^d^	6.5 ± 0.16^d^
	6	8.0 ± 0.20^a^	7.5 ± 0.04^c^	6.4 ± 0.12^d^
	12	7.9 ± 0.06^a^	5.8 ± 0.09^e^	5.9 ± 0.08^e^
T_3_	0	8.1 ± 0.24^a^	6.2 ± 0.05^d^	6.5 ± 0.16^d^
	6	8.2 ± 0.25^a^	7.7 ± 0.35^b^	6.6 ± 0.38^d^
	12	8.1 ± 0.19^a^	5.9 ± 0.27^e^	6.9 ± 0.35^c^
T_4_	0	8.1 ± 0.24^a^	6.3 ± 0.11^d^	6.5 ± 0.16^d^
	6	8.1 ± 0.09^a^	7.4 ± 0.09^b^	6.8 ± 0.18^c^
	12	7.8 ± 0.01^a^	6.8 ± 0.04^c^	7.0 ± 0.11^c^
T_5_	0	8.1 ± 0.24^a^	6.4 ± 0.16^e^	6.5 ± 0.16^d^
	6	8.3 ± 0.34^a^	7.3 ± 0.17^e^	7.5 ± 0.07^b^
	12	8.1 ± 0.26^a^	6.6 ± 0.22^c^	7.8 ± 0.05^a^
T_6_	0	8.1 ± 0.24^a^	6.0 ± 0.18^e^	6.5 ± 0.16^d^
	6	8.2 ± 0.18^a^	8.1 ± 0.31^a^	8.1 ± 0.13^a^
	12	8.2 ± 0.06^a^	8.0 ± 0.06^a^	8.2 ± 0.16^a^
T_7_	0	8.1 ± 0.24^a^	6.2 ± 0.07^e^	6.5 ± 0.16^d^
	6	8.1 ± 0.18^a^	8.3 ± 0.03^a^	8.3 ± 0.20^a^
	12	8.0 ± 0.28^a^	8.1 ± 0.10^a^	8.2 ± 0.14^a^
Standard Ripening 4 °C	0	8.1 ± 0.24^a^	6.5 ± 0.12^c^	6.5 ± 0.16^d^
	6	8.0 ± 0.31^a^	7.4 ± 0.07^b^	8.0 ± 0.41^a^
	12	7.8 ± 0.33^a^	7.6 ± 0.15^b^	8.1 ± 0.06^a^
Positive Control	Ripened at 4 °C or 9 Months	8.1 ± 0.15^a^	8.3 ± 0.48^a^	8.4 ± 0.55^a^

Within a column, means denoted by a different letter are statistically different by Tukey’s HSD Test (*p* < 0.05)

The results presented in Table 5 are the outcome of triplicate treatment and triplicate analysis (3 × 3 = 9; Mean ± SD)

### Limitations

In this investigation, a significant reduction in ripening time of cheddar cheese has been achieved using natural antioxidants. An appropriate mechanism should be adopted to dispose off the ripened/ matured cheese without any further storage. The storage of ripened and matured cheese is not feasible from quality and commercial aspects. Cheese manufacturers who want to adopt rapid ripening of cheddar cheese should establish an appropriate marketing/ dispose off mechanism to avoid unnecessary storage of ripened/ matured cheese.

## Conclusions

An innovation in the accelerated ripening of cheese was achieved. The time required for the ripening of cheddar cheese was reduced half year to 6 weeks only. Sensory characteristics of 6 weeks old cheddar cheese that was ripened at 18 °C was similar to the traditionally ripened chese for 9 monhts at 4 °C. Selenium and vitamin E significantly inhibited the lipid oxidation during accelerated ripening of cheese. Selenium and vitamin E can be used at T_6_ and T_7_ concentrations as antioxidants in accelerated ripening.
